# Complete Genome Sequence of an Ictalurid Herpesvirus 1 Strain Isolated from Blue Catfish (Ictalurus furcatus)

**DOI:** 10.1128/MRA.00082-19

**Published:** 2019-04-11

**Authors:** Kuttichantran Subramaniam, Arun Venugopalan, Andrew J. Davison, Matt J. Griffin, Lorelei Ford, Thomas B. Waltzek, Larry Hanson

**Affiliations:** a Department of Infectious Diseases and Immunology, College of Veterinary Medicine, University of Florida, Gainesville, Florida, USA; b Department of Pathobiology and Population Medicine, College of Veterinary Medicine, Mississippi State University, Stoneville, Mississippi, USA; c Department of Basic Sciences, College of Veterinary Medicine, Mississippi State University, Starkville, Mississippi, USA; d Department of Wildlife, Fisheries and Aquaculture, College of Forest Resources, Mississippi State University, Stoneville, Mississippi, USA; e Thad Cochran National Warmwater Aquaculture Center, Delta Research and Extension Center, Mississippi State University, Stoneville, Mississippi, USA; f MRC-University of Glasgow Centre for Virus Research, Glasgow, United Kingdom; Georgia Institute of Technology

## Abstract

The complete genome sequence of an alloherpesvirus isolated from blue catfish (Ictalurus furcatus) is reported. Genomic analyses revealed that this virus is a distinct strain of ictalurid herpesvirus 1, the first strain of which was isolated previously from a channel catfish (Ictalurus punctatus).

## ANNOUNCEMENT

Ictalurid herpesvirus 1 (IcHV1) is classified in the species *Ictalurid herpesvirus 1*, genus *Ictalurivirus*, and was the first member of the family *Alloherpesviridae* to be sequenced (1). This virus causes acute mortality in young channel catfish (Ictalurus punctatus). Blue catfish (Ictalurus furcatus) are related closely to channel catfish, and channel × blue catfish hybrids are becoming increasingly popular in U.S. aquaculture. In 1998, a population of blue catfish fingerlings reared in an earthen pond exhibited clinical signs like those caused by IcHV1. Moreover, a virus referred to as blue catfish alloherpesvirus (BCAHV) was isolated in the channel catfish ovary (CCO) cell line (ATCC CRL-2772) and exhibited cytopathic effects like those of IcHV1. The partial nucleotide sequence of the gene encoding the DNA polymerase catalytic subunit showed the highest identity (98%, 428/438) to IcHV1 (2).

Clarified CCO cell lysate was centrifuged at 20,000 × *g* for 30 min, and DNA was isolated from the pellet using a Gentra Puregene tissue kit (Qiagen). A DNA sequencing library was prepared using a Nextera XT kit (Illumina) and sequenced on a MiSeq (Illumina) instrument using a v3 600-cycle kit. The 2,027,408 reads were depleted of host (*Ictalurus punctatus*) sequences (GenBank accession no. LBML00000000) using Kraken v2.0 (3) with default parameters. *De novo* assembly of the resulting 1,955,567 reads using SPAdes 3.11 (4) with default settings generated two contigs closely related to the IcHV1 Auburn strain sequence (GenBank accession no. M75136). These contigs were joined into a genome sequence by manually incorporating reads at their ends until they overlapped. The genome termini were identified from large sets of reads commencing at the same positions. The integrity of the sequence was verified by mapping the reads using Bowtie 2.1.0 (5) and inspecting the alignment in Tablet 1.17.08.17 (6) with default settings. The average coverage was 3,883 reads/nucleotide.

The linear BCAHV genome (134,493 bp) consists of a unique region (97,333 bp) flanked by terminal direct repeats (18,580 bp). A total of 91 protein-coding open reading frames (ORFs) were predicted, with 63 in the unique region and 14 in each terminal repeat. These ORFs were identified initially by transferring the IcHV1 annotations to the BCAHV genome using GATU (https://4virology.net/virology-ca-tools/gatu/) with default settings. The annotation was improved by comparisons with other members of genus *Ictalurivirus*, resulting in the substitution of ORF31 and ORF32 by ORF32A, the substitution of ORF40 by ORF40A, and the addition of ORF51A. Three ORFs, namely, ORF16A, ORF57, and ORF62, were predicted from sequence comparisons to be spliced and encode a chloride channel CLIC-like membrane protein, the DNA polymerase catalytic subunit, and DNA packaging terminase subunit 1, respectively.

**FIG 1:**
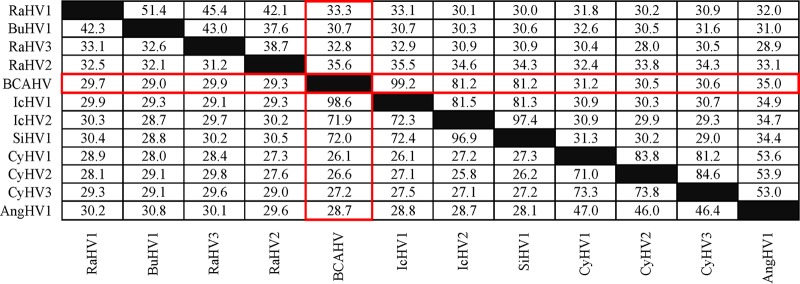
Genetic relationships among alloherpesviruses measured as a percentage of amino acid sequence identity in the DNA packaging terminase subunit 1 (above the diagonal) and DNA polymerase catalytic subunit (below the diagonal) genes. Identities of 100% are shaded in black, and values for BCAHV are outlined in red. Abbreviations: RaHV1, ranid herpesvirus 1; BuHV1, bufonid herpesvirus 1; RaHV3, ranid herpesvirus 3; RaHV2, ranid herpesvirus 2; IcHV1, ictalurid herpesvirus 1; IcHV2, ictalurid herpesvirus 2; SiHV1, silurid herpesvirus 1; CyHV1, cyprinid herpesvirus 1; CyHV2, cyprinid herpesvirus 2; CyHV3, cyprinid herpesvirus 3; AngHV1, anguillid herpesvirus 1.

The BCAHV and IcHV1 sequences are 93.9% identical and colinear except for the absence of ORF16A from IcHV1. An analysis of genetic distances among BCAHV and 11 members or potential members of family *Alloherpesviridae* performed using SDT v1.2 (7), with the MAFFT alignment option implemented, confirmed that BCAHV is most closely related to IcHV1 (Fig. 1). These results indicate that BCAHV may be a novel IcHV1 strain and further expand the known host range of IcHV1.

### Data availability.

The genome sequence of BCAHV (IcHV1 strain S98-675) and sequence read data have been deposited in NCBI GenBank and the Sequence Read Archive under accession no. MK392382 and SRX5493855, respectively.
